# Advances and unmet needs in genetic, basic and clinical science in Alport syndrome: report from the 2015 International Workshop on Alport Syndrome

**DOI:** 10.1093/ndt/gfw095

**Published:** 2016-05-11

**Authors:** Oliver Gross, Clifford E. Kashtan, Michelle N. Rheault, Frances Flinter, Judith Savige, Jeffrey H. Miner, Roser Torra, Elisabet Ars, Constantinos Deltas, Isavella Savva, Laura Perin, Alessandra Renieri, Francesca Ariani, Francesca Mari, Colin Baigent, Parminder Judge, Bertrand Knebelman, Laurence Heidet, Sharon Lagas, Dave Blatt, Jie Ding, Yanqin Zhang, Daniel P. Gale, Marco Prunotto, Yong Xue, Asher D. Schachter, Lori C.G. Morton, Jacqui Blem, Michael Huang, Shiguang Liu, Sebastien Vallee, Daniel Renault, Julia Schifter, Jules Skelding, Susie Gear, Tim Friede, A. Neil Turner, Rachel Lennon

**Affiliations:** 1 Clinic of Nephrology and Rheumatology, University Medicine Goettingen, Goettingen, Germany; 2 Department of Pediatrics, University of Minnesota Medical School, Minneapolis, MN, USA; 3 Department of Clinical Genetics, Guy's and St Thomas’ NHS Foundation Trust, London, UK; 4 Melbourne Health, The University of Melbourne, Parkville, VIC, Australia; 5 Division of Nephrology, Washington University School of Medicine, St Louis, MO, USA; 6 Inherited Kidney Diseases, Nephrology Department, Fundació Puigvert, IIB Sant Pau, Universitat Autònoma de Barcelona and REDINREN, Barcelona, Spain; 7 Molecular Medicine Research Center, Department of Biological Sciences, University of Cyprus, Nicosia, Cyprus; 8 University of Southern California, Children's Hospital Los Angeles, Los Angeles, CA, USA; 9 Medical Genetics Unit, University of Siena, Siena, Italy; 10 Genetica Medica, Azienda Ospedaliera Universitaria Senese, Siena, Italy; 11 Clinical Trial Service Unit and Epidemiological Studies Unit, Nuffield Department of Population Health, University of Oxford, Oxford, UK; 12 Division de Néphrologie, Hôpital Necker, Assistance Publique-Hôpitaux de Paris, Paris, France; 13 Université Paris Descartes, Paris, France; 14 Centre de Référence des Maladies Rénales Héréditaires de l'Enfant et de l'Adulte (MARHEA) Service de Néphrologie Pédiatrique, Clinique Maurice Lamy, Hôpital Necker-Enfants Malades, Paris, France; 15 Alport Syndrome Foundation, Phoenix, AZ, USA; 16 Alport Foundation of Australia, Valentine, NSW, Australia; 17 Pediatric Department, Peking University First Hospital, Beijing, China; 18 University College London-Centre for Nephrology, London, UK; 19 Roche Innovation Center Basel, F. Hoffmann-La Roche Ltd, Roche Pharma Research & Early Development, Basel, Switzerland; 20 Rare Disease Group-Therapeutic Area, Global Clinical Development, Sanofi Genzyme, Naarden, The Netherlands; 21 New Indications Discovery Unit, Translational Medicine, Novartis Institutes for BioMedical Research, Cambridge, MA, USA; 22 Cardiovascular Research, Fibrosis Research, Regeneron Pharmaceuticals, Tarrytown, NY, USA; 23 Clinical Development, Regulus Therapeutics, San Diego, CA, USA; 24 Department of Rare Diseases, Sanofi-Genzyme R&D Center, Framingham, MA, USA; 25 Discovery Biology, Shire, Lexington, MA, USA; 26 Association for Information and Research on Genetic Renal Diseases (AIRG)—France, Paris, France; 27 Federation of European Associations of patients affected by Genetic Renal Diseases, FEDERG, Brussels, Belgium; 28 Alport Israel, Israel; 29 Alport UK, Tetbury, UK; 30 Department of Medical Statistics, University Medical Center Goettingen, Goettingen, Germany; 31 Renal Medicine, Royal Infirmary, University of Edinburgh, Edinburgh, UK; 32 Wellcome Trust Centre for Cell-Matrix Research, Faculty of Life Sciences, University of Manchester, Manchester, UK

**Keywords:** Alport syndrome, chronic kidney disease, guidelines, hereditary kidney disease, nephroprotection

## Abstract

Alport syndrome (AS) is a genetic disease characterized by haematuric glomerulopathy variably associated with hearing loss and anterior lenticonus. It is caused by mutations in the *COL4A3*, *COL4A4* or *COL4A5* genes encoding the α3α4α5(IV) collagen heterotrimer. AS is rare, but it accounts for >1% of patients receiving renal replacement therapy. Angiotensin-converting enzyme inhibition slows, but does not stop, the progression to renal failure; therefore, there is an urgent requirement to expand and intensify research towards discovering new therapeutic targets and new therapies. The 2015 International Workshop on Alport Syndrome targeted unmet needs in basic science, genetics and diagnosis, clinical research and current clinical care. In three intensive days, more than 100 international experts including physicians, geneticists, researchers from academia and industry, and patient representatives from all over the world participated in panel discussions and breakout groups. This report summarizes the most important priority areas including (i) understanding the crucial role of podocyte protection and regeneration, (ii) targeting mutations by new molecular techniques for new animal models and potential gene therapy, (iii) creating optimal interaction between nephrologists and geneticists for early diagnosis, (iv) establishing standards for mutation screening and databases, (v) improving widespread accessibility to current standards of clinical care, (vi) improving collaboration with the pharmaceutical/biotech industry to investigate new therapies, (vii) research in hearing loss as a huge unmet need in Alport patients and (viii) the need to evaluate the risk and benefit of novel (including ‘repurposing’) therapies on an international basis.

## INTRODUCTION

The 2015 International Workshop on Alport Syndrome (AS) was held at the Old Gauss Observatory in Göttingen, Germany from 25 to 27 September. As a continuation of the successful concept of the previous workshop in January 2014 [1], this meeting covered clinical, genetic and basic science aspects of AS. More than 100 clinicians, researchers, patient organizations and pharmaceutical/biotech industry representatives from all over the world intensively discussed various topics. The overall goal of the meeting was focussed on strengthening worldwide research strategies and collaborations and on developing and evaluating new and existing treatments.

## KEYNOTE PRESENTATIONS FROM PARALLEL RESEARCH AREAS

Charles Streuli, a cell and matrix biologist from Manchester, UK, presented new concepts relating to the regulation of extracellular matrix (ECM) and how this can influence cell fate. These included the mechanical properties of tissue and how ageing- or disease-associated changes in tissue stiffness can predispose to cancer. The concept that tissue stiffness can influence cell fate is not new, and indeed it is known that stem cells differentiate to different lineages (adipose or bone) depending on ECM stiffness [2]. However, the association between tissue stiffness and the development of cancer is more recent [3]. Streuli also introduced the concept of the body clock and the regulation of the ECM. There is growing evidence that circadian rhythms influence a range of physiological processes and the ECM is no exception [4]. The relevance of these observations for patient management may be through the timing of drug therapies, as recently demonstrated in a model of inflammatory lung disease [5].

Alexander Nystrom, a group leader at the Klinik für Dermatologie und Venerologie, Freiburg, Germany, talked about the skin disorder dystrophic epidermolysis bullosa (DEB). As with AS, this devastating skin disease is caused by genetic mutations in a collagen gene, *COL7A1* [6]. Collagen VII is important for tight attachment of the dermis to the epidermal basement membrane, and loss of function results in a blistering skin disorder with increased propensity for tissue scarring. Proposed treatment options for patients with DEB have included bone marrow transplantation, collagen VII protein replacement therapy, gene therapy, fibroblast therapy and induced exon-skipping therapy [7]. A number of these approaches have also been considered for AS. Nystrom further described the creation of a mouse model of DEB, and in this model, transforming growth factor (TGF)-beta was up-regulated in wounds. Treatment with the angiotensin II receptor inhibitor (ARB) losartan was associated with reduced scarring, suggesting an anti-fibrotic role for this therapeutic agent. In keeping with a role for tissue mechano-biology, this study also reported a softer dermis after extended losartan treatment [8].This example of repurposing the use of existing therapies allows for accelerated clinical impact.

The AS is characterized by abnormal ECM accumulation in the form of glomerulosclerosis and ultimately tubulointerstitial fibrosis. Michael Zeisberg, a group leader and clinician scientist from Göttingen, Germany, gave a keynote talk on the role of epigenetics in renal fibrosis. He described the role of Rasal1 as an epigenetic modifier that inhibits the Ras-GTP oncoprotein [9]. This study found hyper-methylation of RASAL1 by the methyltransferase Dnmt1 and that gene reduction of Dnmt1 in a mouse model was associated with reduced renal fibrosis. Zeisberg's team have gone on to demonstrate that free DNA fragments of the promoter region of RASAL1 can be detected in peripheral blood and that these levels correlate with RASAL1 levels in kidney tissue and in turn with the extent of fibrosis [10]. In the same study, the authors found that RASAL1 DNA levels were reduced following treatment with low dosage of the anti-hypertensive drug dihydralazine. Therefore, this candidate represents a potential new biomarker for renal fibrosis and perhaps even a new therapeutic target.

Providing great insight into the bench to bedside pathway for gene therapy Stephen Hyde, Head of the Oxford University Gene Medicine Research Group, gave another keynote presentation. After the discovery of the first gene defect in cystic fibrosis (CF) in 1988, Hyde described the race to develop a cure with gene therapy. This started with the generation of a mouse model in which it was established that gene transfer would be possible [11]. There were then numerous combinations of genetic constructs coupled with a delivery system to establish the most efficient transfer combination. Ultimately, these efforts resulted in the development of the CF consortium in the UK where research teams from three leading institutions (Edinburgh University, Imperial College London and University of Oxford) combined efforts to launch the first clinical trial. This Phase II trial was recently reported and demonstrated a modest improvement in lung function in CF patients [12]. The trial recruited an unselected cohort of patients and in addition, during the trial development, a new small-molecule therapy became available to a small subgroup of CF patients. Hyde discussed the importance of patient selection and indicated that further improvement in gene therapy would be possible with the use of viral vectors for gene delivery.

Small-molecule therapy options are also being considered for AS, and a Phase II clinical trial of the anti-fibrotic agent targeting microRNA-21 (miR-21) is currently underway. Markus Bitzer, a nephrologist and group leader at the University of Michigan, USA, provided further insight into the actions of miR-21. An uncontrolled RNA-sequencing study in PIMA Indians (who are especially prone to developing diabetic nephropathy) found some correlation in that glomerular miR-21 was very abundant [13]. miR-21 was also increased in a mouse model of diabetic kidney disease, and loss of miR-21 in these mice accelerated the progression of kidney disease [13]. The authors proposed that miR-21 is an important regulator of TGF-beta, itself a major player in fibrogenesis. Bitzer also described the NEPTUNE consortium, established for the study of nephrotic syndrome, and highlighted the importance of links with the pharmaceutical/biotech industry for the progression of research in this area.

The wider therapeutic targets for AS were considered by Hans Joachim Anders, nephrologist and group leader from Munich, Germany. He reminded us of the need to consider the number of nephrons a patient has from birth as this may ultimately impact upon the severity of genetic or acquired renal disease. Birth weight is a strong predictor of nephron number [14], and whilst regeneration possibly exists, Anders explained that this is likely to represent a very small component of renal growth with a much greater contribution from nephron hypertrophy. With regard to therapies for AS, Anders described the potential for multi-drug usage. A recent meta-analysis of combination therapy to lower blood pressure in diabetic kidney disease demonstrated once again that angiotensin-converting enzyme (ACE) inhibitors and ARBs were the most effective agents at prolonging renal survival but that combination therapy needed to be balanced against the risks of hyperkalaemia and acute kidney injury [15]. The effect of the mineralocorticoid antagonist finerenone was also discussed, and a recent clinical trial in diabetic kidney disease found reduced albuminuria in patients treated with a combination of ACE inhibitors or ARBs and finerenone [1]. These clinical trial findings may be relevant for strategies to treat AS to delay the progression of renal disease.

## UNMET NEEDS IN RESEARCH: REPORT OF THE BASIC SCIENCE BREAKOUT GROUP

The workshop also provided the opportunity for small group discussions, followed by panel discussion, with feedback from the whole audience. In the basic science group, a number of unmet needs were identified. The need to establish an ‘International Alport Syndrome Research Alliance’ to provide legitimacy to our individual efforts was raised. In addition, the need for an international biobank was discussed and envisaged to be in collaboration with patients and clinicians, and to provide material (blood, urine and tissue samples) for state-of-the-art analyses of disease progression, as well as material to validate findings from mouse models. Possibilities for maintaining research tools, including human cell lines, antibodies, plasmids, mouse colonies, etc., were discussed with the final goal of facilitating sharing among members of the AS research community. The accessibility of the biobank by pharmaceutical/biotech industry would need to be sensitively explained to patients. The need to harness the power and knowledge of developmental and stem cell biology and regenerative medicine to improve research tools and to aid discovery was highlighted. The participants also agreed that it is important to increase our knowledge about human kidney development in order to understand podocyte specification and glomerular basement membrane (GBM) deposition. In terms of new therapies, a focus on methods to strengthen or supplement the existing collagen IV α1, 1 and 2 network to slow the decline in kidney function was considered. In addition, the feasibility of expressing α3/4/5 in endothelial cells by viral transduction was raised, as was consideration of molecular techniques to correct glycine substitutions and nonsense mutations (such as induced stop codon skipping). Given the presumed central role of podocytes, a focus on assessing whether foot process effacement and loss of podocytes could be halted through inhibition of pro-fibrotic, pro-apoptotic or aberrant collagen-induced signalling was raised, as was the question of whether protein folding and/or endoplasmic reticulum stress could be ameliorated with chemical chaperones or other small molecules.

The group acknowledged the need for the pharmaceutical/biotech industry's expertise to help direct research efforts towards achievable therapeutic strategies. The ultimate need for precision medicine was also discussed and in particular the need to identify modifier genes and use their activities as potential therapeutic targets. The advent of next-generation sequencing technology now offers the possibility of translating this goal into reality, but large cohorts of patients stratified on the basis of detailed molecular and clinical characterizations are needed. As demonstrated by the Italian group, genes of the ECM or podocyte cytoskeleton represent modifier candidates that can be investigated by exome/genome panels before an unbiased data analysis.

Finally, the need to attract researchers into under-represented areas of research in AS was raised, and the example of research in hearing impairment was high on this agenda.

## UNMET NEEDS IN DIAGNOSIS: REPORT OF THE GENETICS & DIAGNOSIS BREAKOUT GROUP

The Genetics & Diagnosis breakout group sessions (and a special pre-meeting) discussed late-breaking achievements and developments in the fast-growing field of molecular genetics extensively in the context of earlier and more accurate diagnosis. Why is this issue so important? It is highly likely that the current EARLY PRO-TECT Alport trial of early ACE inhibition (www.clinicaltrials.gov; identifier NCT01485978) with ramipril [16] will be followed by other trials as candidate therapies emerge from basic science research or from other clinical trials [17]. It is thus essential that all patients who enrol in these trials have their diagnosis confirmed at the DNA level so that any possible genetic factors that may influence response to therapy are identified. The group discussion covered the most recent reviews and expert recommendations about five different topics.

### Achieving an early diagnosis: the role of geneticists and nephrologists

There is considerable evidence that early diagnosis and treatment, if indicated, improve the long-term prognosis in AS [18, 19], and this evidence forms the basis for treatment recommendations [20, 21]. In order to achieve earlier diagnosis, there needs to be greater awareness of the possibility of a diagnosis of AS in any patient presenting with persistent haematuria. In childhood, this is more likely to be achieved if any child with unexplained, persistent haematuria is referred to a paediatric nephrologist. Genetic testing is generally replacing more invasive investigations such as kidney biopsy and skin biopsy and is likely to achieve confirmation of the diagnosis at a molecular level in around 95% of patients who have AS [21]. In some families where the diagnosis has already been made in relatives, parents may request genetic testing at birth, and whilst this is not urgent from a clinical point of view, it is possible if the mutation is known and certainly should be considered by around the age of 5 years in order to ensure regular monitoring and early treatment of any proteinuria or hypertension that may develop: enrolment to early intervention trials also becomes an option. The use of skin biopsy varies in different parts of the world and is generally more prominent in America as it is cheaper than genetic testing. In Israel, a positive skin biopsy is required to get reimbursement for genetic tests. In China, skin biopsy is well accepted as it is less invasive and cheaper. Elsewhere skin biopsies are less likely to be used for diagnostic purposes as the identification of a specific *COL4* mutation is more useful.

### Minimum standards for collagen type IV mutation screening

Sanger sequencing used to be the gold standard, but now most laboratories use next-generation sequencing to screen the genes corresponding to the α3, 4 and 5 chains of type IV collagen simultaneously. This is now the gold standard not only for the speed of the analysis, but also because it ensures the detection of the digenic forms, i.e. those forms due to mutations in two different type IV collagen genes. This will detect most point mutations, but copy number variation analysis is also required; otherwise, 15% of mutations in the X-linked gene and a smaller percentage of autosomal mutations may be missed. If next-generation sequencing is negative, then clinicians may resort to kidney biopsy (with electron microscopy and immunostaining), skin biopsy, RNA analysis (obtained from skin or hair root) or further genetic testing. Renal panels that can be screened by whole-exome sequencing are being developed, and in some patients who were initially thought to have AS, mutations in other genes expressed in the kidney may be identified. There are clear European and American guidelines for determining the pathogenicity of variants, and most laboratories participate in external quality assessment schemes such as the European Molecular Quality Network. The value of submitting mutations to databases, together with relevant clinical information, is undisputed, but it is challenging in practice because of the resources required to do this. Clinicians (and patients) are strongly advised to ensure that the testing services they use are comprehensive and undertaken in accredited labs that meet relevant ISO standards, offering in-house validation of potentially pathogenic variants that are identified.

Mutations in other genes can also affect the phenotype of patients who have at least one mutation in a type IV collagen gene. These include *MYH9*, *CFHR5* and *NPHS2*, among others [22]. A novel *COL4A1* frameshift mutation was demonstrated to cause kidney disease without extra renal involvement in a large Turkish Cypriot family (see Figure 2). Several groups have now published examples of digenic inheritance in AS, and as gene panels are more widely used, this list will expand.

### Providing a map of genetic mutation screening services

There are a number of useful online resources, including Gene Tests and the UK GTN website. Orphanet lists some labs, but is not completely up to date. The main reason that not all patients are offered genetic testing is primarily due to the cost of these tests, although if they enable more invasive procedures such as renal biopsy to be avoided, the net difference may be less significant. Providing access to genetic testing in developing countries continues to be a challenge. The tests are expensive, and unfortunately countries where genetic testing is not available/affordable are likely to continue to see higher rates of renal biopsy and skin biopsy. Genetic tests have advantages over other forms of testing as they make the diagnosis of AS with certainty, even in females, who only have haematuria; they also clearly identify the mode of inheritance which is important for counselling and may help predict the clinical course. 

### Easier (and earlier) diagnosis of Alport syndrome in everyday clinical practice: possible role of a new classification system

A major requirement for the timely treatment of AS is to make early diagnosis (of this rather complex type IV collagen disease with various inheritance paths) easier in everyday clinical practice. Heterozygous individuals at risk of renal failure are often underdiagnosed. The value of a new, less complex and broader classification system for all patients who have a *COL4* mutation (including those previously labelled as ‘familial benign haematuria’) was discussed. There are concerns about causing distress for the parents of a child with isolated haematuria, but labelling all patients with a *COL4* mutation as having AS may help to ensure that all patients at risk of developing significant renal disease are offered proper lifelong surveillance and treatment. There is complete agreement as to the need for at least annual monitoring of blood pressure and screening for proteinuria for all mutation carriers. The discussion about a new classification system for AS continues.

### Prenatal diagnosis and pre-implantation genetic diagnosis

Couples who know that they are at significant risk of having a child affected with AS may express an interest in prenatal or pre-implantation genetic diagnosis. This is becoming widely available in countries where the law permits these reproductive options, although demand varies. These tests should only be discussed and offered in the context of genetic counselling if there is complete confidence at a clinical and genetic level of the diagnosis including the distinction between monogenic and digenic forms.

## UNMET NEEDS IN CLINICAL CARE: REPORT OF THE CLINICAL CARE AND REGISTRIES BREAKOUT GROUP

A diverse group of paediatric and adult nephrologists, clinical researchers and patient advocacy group representatives convened in breakout sessions over 2 days to discuss priorities for research into the clinical aspects of care for patients with AS.

The current treatment of chronic kidney disease (CKD) in children and adults with AS was reviewed. There are currently no approved therapies specifically designed for AS-related kidney disease. The group members agreed that the current clinical practice recommendations for treatment with ACE inhibitors (and angiotensin receptor antagonist as second line) for proteinuric individuals in low-risk categories and microalbuminuric individuals in high-risk categories remain consistent with current evidence [20]. The ongoing EARLY PRO-TECT Alport trial is a double-blind, randomized, placebo-controlled trial of paediatric patients with isolated haematuria or microalbuminuria comparing disease progression over 3–5.5 years in subjects treated with ramipril versus placebo [16]. The results of this trial may influence treatment recommendations for children with early-stage AS in the near future. Whilst there are retrospective data from the European Alport Registry to suggest that ACE inhibitor therapy delays renal failure and improves lifespan, there was agreement that this analysis should be replicated in other registries around the world including large numbers of patients to ensure that it is a consistent finding [18, 19]. Using additional drugs to lower urinary protein excretion, beyond that achieved by full-dose ACE inhibition, is not of proven benefit and may convey additional risk. Further studies in AS and other proteinuric renal diseases are required before additional drugs can be recommended. Participants agreed that care of CKD in children and adults with AS should follow established KDIGO guidelines [23]. Currently, there is no evidence suggesting that individuals with AS should have different blood pressure, anaemia, cholesterol, parathyroid hormone, etc., targets than the general CKD population.

The group identified that a major understudied problem in AS is the hearing loss that occurs in a majority of affected individuals. Hearing is normal at birth followed by the development of high-frequency sensorineural hearing loss in 50% of affected males with X-linked Alport syndrome by the age of 15 years and 90% by the age of 40 years [24]. Adults with hearing loss are less likely to complete higher education and may be more likely to experience depression and career dissatisfaction [25, 26]. Children and adolescents with hearing loss may be further impacted by teasing and poor interaction with peers [27]. Children and adults with hearing loss and AS are at risk for lower quality of life measures; however, this has not been evaluated in AS patients specifically and may be exacerbated by their concomitant CKD. Approaches to identify and treat the psychosocial aspects of hearing loss in this population are needed.

Strategies to preserve hearing in patients with AS are also needed. There is some information available about the distribution of type IV collagen chains in the inner ear in animal models of AS as well as in humans [28–31]. The structural changes observed in human Alport cochleae may be associated with defective attuning of basilar membrane motion and hair cell stimulation, resulting in reduced acuity of hearing, although the definitive mechanism for the development of hearing loss is unclear. Studies of hearing and the effect of therapy on hearing loss in animal models of AS should be performed. Future clinical trials should include changes in hearing as an outcome measure if there is any biologically plausible evidence that a new treatment could modify the hearing loss. In addition, the Alport community needs to encourage interest and research in AS-related hearing loss in the otolaryngology and audiology communities via targeted grant opportunities and educational seminars.

AS is a disease of families, often with multiple affected family members. Research into the psychological aspects of every stage of the disease course is needed, including support at diagnosis and transition from paediatric to adult providers. Research is also needed on how to support individuals and families through stages of hearing loss, CKD, dialysis, transplantation and family planning. Participants agreed that psychosocial support is generally lacking for all children and adults with CKD and that this should be an area of research focus in the nephrology community. Quality of life measures should be included in any clinical trials for AS. As AS is a rare disease, diagnosis-specific resources are most likely to be useful when provided remotely via the internet, ideally translated into several languages. Several Alport patient advocacy groups have developed their own methods for patient and family support. The Alport Syndrome Foundation (USA) offers both public and private Facebook pages offering advice and fellowship. The Italian Alport association has started a project on ‘recognize Alport syndrome’ on its webpage and distributed a number of informative brochures in key Italian centres. Alport UK have launched a study with University of Oxford to document personal experiences of living with the AS. The videos and audio material will be featured on the award-winning website healthtalk.org later in 2016. The AS has a broad geographic distribution around the world without concentration in any one geographic or ethnic region. Strategies to support and promote the standard of care for AS patients in developing countries are needed. In developing countries, access to genetic testing and renal biopsy may not be widely available; however, urine dipstick and ACE inhibitors are relatively inexpensive. The International Paediatric Nephrology Association has sponsored an AS symposium at each of their last several triennial meetings to increase awareness of AS and treatment recommendations for clinicians from developing countries. We would support initiatives through the International Society of Nephrology to do the same for adult nephrologists.

People with AS often do well following renal transplantation, with graft and patient survival that is better than the general end-stage renal disease population; however, they are at risk of anti-GBM disease post-transplant due to formation of autoantibodies [32, 33]. The incidence appears to be lower than previously reported, with only 0.4% of patients affected in a recent retrospective review [34]. Evidence to guide recommendations for monitoring and treatment of anti-GBM disease is not available, and this topic deserves further study.

The group considered an international clinical registry to be an important milestone in developing clinical research in AS further. A working group that will concentrate on developing the International Alport Registry was formulated. Such a registry could potentially provide data on the epidemiology and natural course of AS. Furthermore, it could be used to recruit patients into clinical trials, and its data could support small clinical trials in a generalized evidence synthesis approach. Smaller Phase I and IIa/proof of concept (PoC) trials of new investigative compounds will also rely on validated pharmacodynamic and clinical efficacy markers. Target-specific pharmacodynamic markers enable faster selection of effective doses in fewer cohorts of healthy volunteers in Phase I. Sensitive, specific and ideally non-invasive efficacy markers enable smaller, shorter Phase IIa PoC trials in rare diseases such as AS.

## SUMMARY AND NEXT STEPS

**FIGURE 1:**
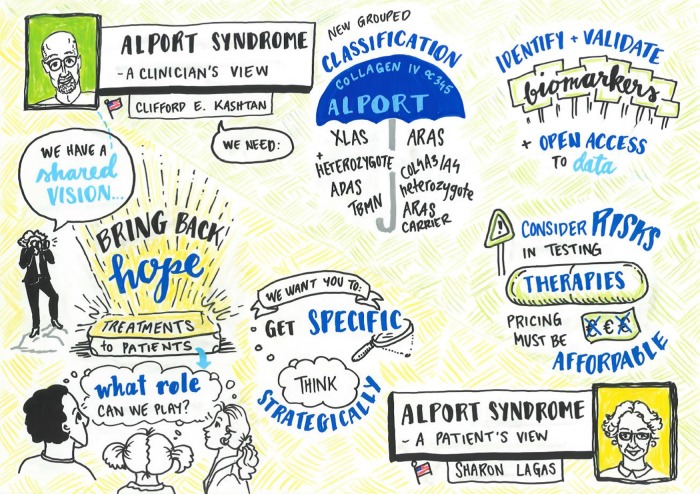
Extract of some of the most important clinical topics discussed at the workshop. Some of the most relevant clinical aspects and unmet needs were addressed by Clifford Kashtan (clinician's view) and Sharon Lagas (patient's view): (i) the value of a new, easier classification of the AS to enable earlier diagnosis and treatment; (ii) the role of biomarkers, international registries and open access to data; (iii) possible risks (and pricing) of new therapies; (iv) combined effort of researchers, clinicians, patients and the pharmaceutical/biotech industry to find better therapies; and (v) the role of patient organizations. XLAS, X-linked Alport syndrome; ADAS, autosomal dominant Alport syndrome; TBMN, thin basement membrane nephropathy; ARAS, autosomal recessive Alport syndrome.

The 2015 International Workshop on Alport Syndrome continued the successful concept of the workshop in January 2014 [1]. This meeting was organized through the concerted efforts of patient advocacy groups from four continents. After a very warm welcome by the organizers, the Dean of the University Medical Center Goettingen and the German patient organization, all delegates expressed their specific interests in the clinical, genetic and basic science aspects of AS. Importantly, the workshop concentrated on the most essential unmet needs in AS, as addressed by Clifford Kashtan (clinician's view) and Sharon Lagas (patient's view) (Figure 1). A separate publication will address and discuss the requirement to develop a better understanding of the impact of AS on women and girls and relevant treatment.

**FIGURE 2:**
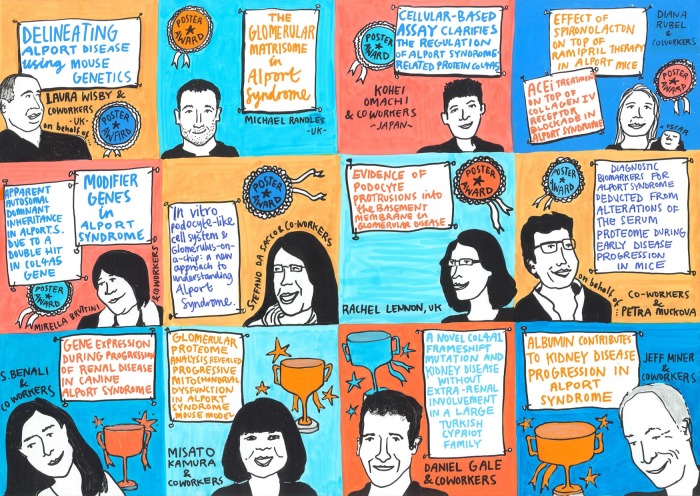
Poster prizes and young investigator awards of the 2015 International Workshop on Alport Syndrome.

More than 30 posters were presented and hotly debated in panel sessions, thereby contributing significantly to the conclusions of the breakout groups. Eight poster awards and four young investigator awards were handed over to the best presenters (Figure 2).

**FIGURE 3:**
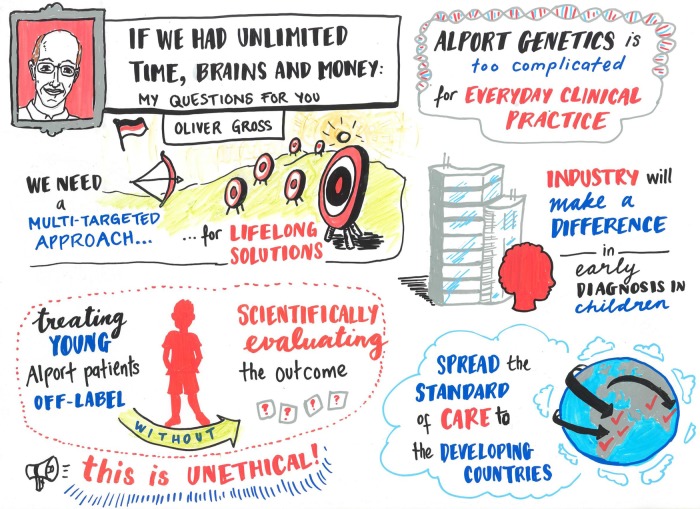
Extract of urgent unmet needs validated in panel discussions. Some of the most relevant unmet needs were: (i) the need for a multi-target, flexible, patient-specific individual approach for lifelong therapy of AS to minimize the progress of disease; (ii) the need for a less complicated, straight forward diagnosis; (iii) the need to scientifically evaluate the outcome of treating young patients on a broad international basis; (iv) chances to spread the standard of care to developing countries; and (v) the urgent need for new therapies, as industry will make a difference in the awareness of AS as a treatable disease that requires early diagnosis.

A ‘lock-in’ environment provided an internationally diverse group of physicians, geneticists and scientists from academia and the pharmaceutical/biotech industry, many of whom were not specifically AS experts, with opportunities for intense, open-minded discussions that led to major conclusions. Urgent unmet needs were addressed (Figure 3) and validated in panel discussions. The next steps to follow in early 2016 are (i) further discussion on whether changes to the nomenclature for AS might assist in earlier diagnosis and (ii) large-scale international funding applications for a multi-target approach from the basic and clinical research consortium to investigate diverse aspects of AS.

Building upon accomplishments from 2014, the 2015 International Workshop on Alport Syndrome further consolidated interactions among the various stakeholder groups and addressed upcoming steps to move forward in the shared mission to find a long-term multi-target therapy for AS resulting in a lifelong delay of renal failure or even a cure.
